# Functionality Pattern Matching as an Efficient Complementary Structure/Reaction Search Tool: an Open-Source Approach

**DOI:** 10.3390/molecules15085079

**Published:** 2010-07-27

**Authors:** Norbert Haider

**Affiliations:** Department of Drug and Natural Product Synthesis, University of Vienna, Althanstraße 14, A-1090 Vienna, Austria; E-Mail: norbert.haider@univie.ac.at; Tel.: +43-1-4277-55624; Fax: +43-1-4277-9556.

**Keywords:** open-source, checkmol, matchmol, functional groups, structure databases

## Abstract

An open-source software package for creating and operating web-based structure and/or reaction databases is presented. Besides standard search capabilities (text, structure/substructure/similarity), the system offers a fast additional search option, entirely based on binary pattern matching, which uses automatically assigned functional group descriptors.

## 1. Introduction

The management of compound archives usually requires a carefully designed and well-maintained compound database system. Web-based solutions are very attractive for such a purpose, as they can offer all or parts of the available information to a broader public without requiring the installation of dedicated software on the client side. The NIH/NCBI PubChem database may serve as a typical example of such a web-accessible chemical compound database with full support of structure/substructure search [1]. This very large collection of structures and associated data currently contains more than 40 million entries; and whose cheminformatics infrastructure was built around OpenEye’s OEChem library [2] and the Cactvs toolkit [3,4]. Various other software products suitable for running a web-based chemical structure database are available, such as the JChem package from ChemAxon [5], but most of these solutions are commercial products and thus closed-source applications. Among the open-source projects, the Chemistry Development Kit (CDK) [6] deserves particular attention, as it provides a powerful and flexible framework of Java routines which can be integrated into various kinds of cheminformatics applications, including web services. Other promising open-source approaches aim at the extension of general-purpose database management systems like MySQL [7], PostgreSQL [8], or Oracle [9] with add-on modules providing structure/substructure search functionality and other features of “chemical intelligence” by extending the syntax of the Structured Query Language (SQL), e.g., via integration of the Open Babel chemistry toolbox [10]. An overview of these projects like Pgchem [11] can be found at the ChemiSQL Wiki website [12]. By nature, all of these toolkits/frame­works/add-ons require some amount of programming in order to assemble them into a fully functional website with structure search capabilities. While this software integration effort is easily feasible in institutions with sufficient cheminformatics expertise and/or software development/support personnel, it may pose a problem for smaller units (e.g., experimentally oriented chemistry research groups) wishing to share their small to medium-sized collections of chemical structures via a website. With this need in mind, a software package was developed and made freely available which is easy to install and manage, but offers all of the essential search options. In the following, the principal features of the current release of this package, MolDB5R [13], will be described and its special capabilities for handling information about functional groups in molecules will be discussed in more detail.

## 2. System Description

### 2.1. General Concept

**Figure 1:**
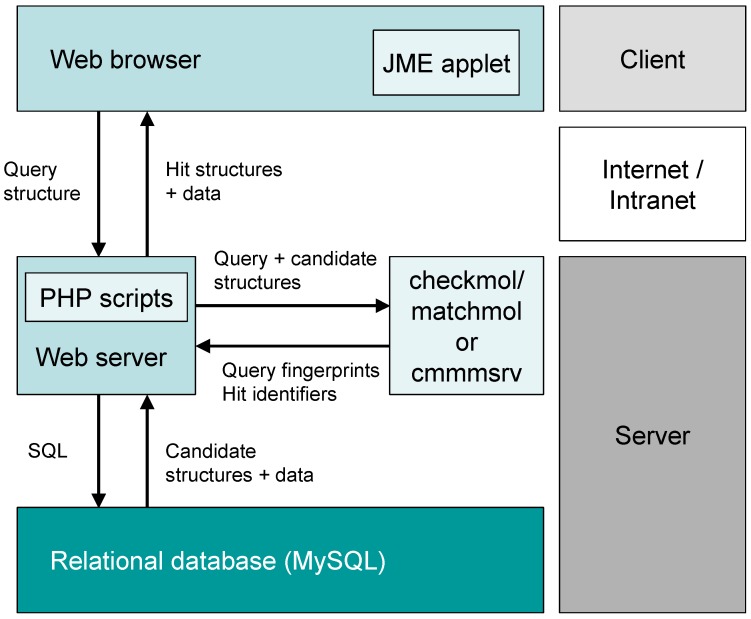
Client/server architecture of MolDB5R.

The MolDB chemical database software was developed as a compact application package which runs on top of a combination of very common hard- and software. As hardware, any industry-standard x86 PC can be used and Linux was chosen as the primary operating system (MS Windows is also supported with some limitations). Web server functionality is provided by the popular Apache software [14] in combination with the PHP scripting language [15]. For storage of chemical structures (as molfiles) and associated textual and numeric data, the MySQL relational database management system [7] is used. All of these components are open-source software and they are included in most Linux distributions. The MolDB5R package (see Figure 1) consists of a set of PHP scripts which provide the user interface for the various search options. The central element for chemical structure/ functionality analysis and structure comparison is provided by the open-source program, checkmol/ matchmol [16] which will be described in more detail below. For setup and administration of the system, a collection of utility programs written in the Perl scripting language is included. For instance, there are scripts for batch import and export of compound data in the commonly used MDL SD (structure+data) or RD (reaction+data) file formats [17]. As a tool for input and (to some extent) display of chemical structures on the web pages, the JME Java applet is used, which is freely available from its author, P. Ertl [18,19]. The main features of the MolDB5R package are:

- text search in structure and reaction data collections
- structure/substructure/similarity search in structure data collections
- structure/substructure search in reaction data collections
- functional group search in structure and reaction data collections
- administration front-end for entering/editing structures or reactions and associated data

For structure/substructure search operations, a typical two-stage process of efficient pre-selection and subsequent atom-by-atom comparison (subgraph isomorphism) of the query structure with the candidate structures is used. The checkmol program is employed for the generation of pre-selection descriptors (fingerprints) whenever a new structure or reaction is entered into the database, while its companion program, matchmol, is responsible for the final structure-matching operation.

### 2.2. Analysis of Chemical Structure and Functionality with Checkmol

With its origin in an educational software project, the checkmol program has grown into a multi-purpose tool which offers a variety of analysis functions. It is a compact command-line program written in the Pascal language. It reads chemical structures in MDL molfile format [17] either from a disk file or from standard input, the results are always written to standard output (*i.e.*, to the screen in a text-based console). Depending on command-line options specified, output can be as follows:

- a list of all detected functional groups in clear text (English or German)
- the same list as a bitstring (in 32-bit segments, each represented as unsigned integer number)
- the same bitstring in ASCII representation (*i.e.*, as a string composed of “0” and “1” symbols); in both representations, each position in the bitstring represents the presence or absence of a particular functional group in the molecule
- a list (in one of two possible output formats) of discrete structural descriptors (“molecular statistics” = “molstat”), useful for pre-selection in a structure database
- a list (in one of two possible output formats) of hash-based fingerprints, useful for pre-selection in a structure database
- the chemical structure in MDL molfile format with additional encoding for aromaticity

**Figure 2:**
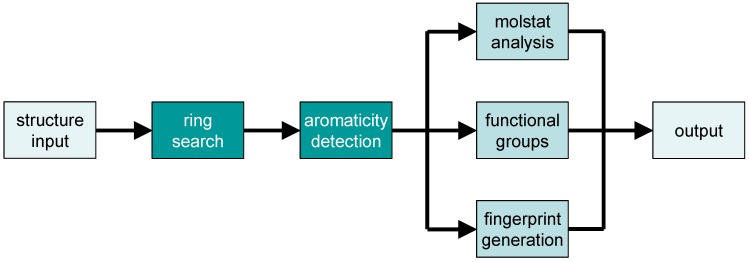
Main workflow for analysis of a chemical strucure by the checkmol program.

Whereas some parts of the program are always involved, such as ring search and aromaticity detection, other subroutines are only invoked on demand, depending on the command-line options which have been specified (see above; some of these options can be combined). A typical workflow is depicted in Figure 2.

In order to reliably detect all aromatic structures, a ring search is performed, using the set of all rings (SAR) [20] as the default ring set (with a fallback option to a smaller ring set for highly con­densed systems). These rings are then examined for their aromatic character, applying the following criteria: the Hückel rule of 4n+2 π electrons must be met and conjugation must not be interrupted. Charged atoms (like in the cyclopentadienyl anion or in the tropylium cation) and hetero­atoms with free electron pairs (like in furan) are considered as well as mesomeric structures contributing to aromaticity (like in 2- or 4-pyridones). As these detection routines require some computational effort, it appears reasonable to perform them only once during entry of a new structure into a database and to store this information permanently in a suitable manner. For this purpose, checkmol can generate a slightly modified (“tweaked”) molfile output in which aromaticity information for atoms and bonds is contained transparently in unused columns of the tabular molfile data structure. After completion of aromaticity detection, all atoms of a molecule are classified into subtypes (in addition to elemnt, iso­tope, charge, and radical character as specified in the input), based on their state of hybridization.

Depending on the command-line options, the program then branches into different tasks of further analysis. Discrete numeric descriptors (“molstat”) are generated by collecting quantitative information about certain structural elements by examination of the atom and bond lists in the connection table and of the internal ring list. These descriptors include the number of atoms, bonds, rings, carbon atoms (any type), carbon atoms (sp, sp^2^ hybridized), carbons bonded to one/two/three or more heteroatom(s), C-O fragments, C=O fragments, C/N fragments (any bond type), rings of a particular size (3/4/5/6/7/8/9/10/11/12/13 or more), rings containing N/O/S, *etc*. [21]. 

For the generation of hash-based binary fingerprints, which are a commonly used criterion for structure pre-selection in databases [22,23,24], the molecular graph is disassemled into all possible linear fragments with a length ranging from three to eight atoms. Strings representing atom types as well as bond types of these linear fragments are then passed to two independent hash functions (taken from the publicly available General Purpose Hash Function Algorithms library [25]) in order to compute two pseudo-random numbers in the range of [1-512]. A bitstring with a length of 512 bits is used to represent the binary fingerprint of the molecule. Initially, this bitstring contains only “0” bits, the two numbers generated for each possible linear fragment indicate two positions in this bitstring which are set to “1”. In a test set of one million structures which were taken from the PubChem database, this algorithm produces fingerprint bitstrings with an average bit density (“darkness” [26]) of approximately 35%, which represents a good compromise between selectivity and memory usage. These bitstrings can be conveniently handled and stored as 32-bit segments, represented by the corresponding 32-bit integer numbers (unsigned).

As a special feature of checkmol, chemical structures are also characterized by means of another set of binary descriptors which represent the absence or presence of particular functional groups in the molecule. For this purpose, the program analyzes the molecular graph by going through the atom list and bond list and by calling various subroutines, depending on atom and bond types. For instance, when a double bond is encountered, the function chk_double() is invoked. Within this function, the number of valences between carbon and connected heteroatoms is counted and – if e.g., it is found to equal 3 – another subroutine chk_carboxyl_deriv() is called. The latter function inspects all heteroatoms (including their substituents) attached to the reference carbon and then assigns the appropriate functional group such as ester, acid chloride, amide, amidine, *etc*. by setting the bit value at a certain position in the bitstring to “1”. The current version of checkmol is able to recognize about 200 functional groups, an illustrated list containing the position numbers in the functional group bitstring has been published [27].

**Figure 3:**
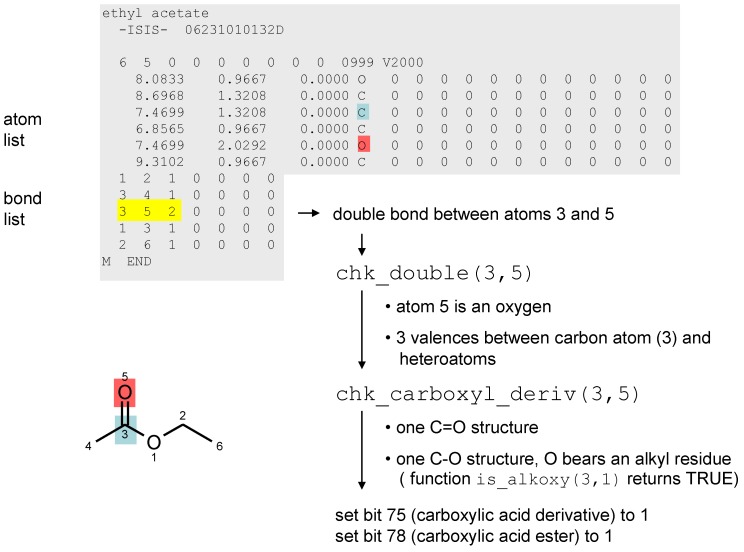
MDL molfile of ethyl acetate, pseudo-code for detection of the ester group.

All of these molecular descriptors are generated whenever a new molecule is entered into the database and they are stored together with the structure and other textual and numeric data in dedicated database tables. Molecules can be entered either interactively via a web front-end (which uses the JME applet for drawing) or via batch processing of SD files with the Perl script, sdf2moldb.pl, which splits the SD file into its records, invokes checkmol, and stores the various parts of information in the corresponding database tables. For chemical reactions, which also can be entered either interactively or by batch import from RD files, cumulative fingerprints for all reactants and for all products are computed and stored. The molecular statistics descriptors are not used in the case of reactions.

Both for structure and reaction collections, another set of fingerprints is optionally generated by comparison of the input structure(s) with a pre-defined dictionary of structural elements (fragments). By default, these dictionary-based fingerprints are derived from a collection of 62 common carbocyclic and heterocyclic ring systems, but the dictionary is exchangeable and/or expandable. Structural comparison is performed by matchmol, the companion program of checkmol (see below).

### 2.3. Structure Matching with Matchmol

The matchmol program expects at least two chemical structures as input in molfile format, treating the first one as the query structure (“needle”) and any following structure as a candidate (“haystack”). By default, output is the consecutive number (starting with 1) of the candidate structure and a flag (T or F) indicating whether or not the query structure represents a substructure of this candidate. Controlled by command-line arguments, the search can be restricted to exact matches and the program can be forced to consider geometrical isomers (*E/Z* isomer discrimination) and asymmetric centers (R/S isomer discrimination). In the latter case, chiral centers can be represented in the input structures either with true 3D (XYZ) coordinates or with “up” or “down” bond symbols in 2D molfiles.

In fact, checkmol and matchmol are the same executable program, but they behave differently depending on the name by which the program is invoked. Whenever matchmol encounters an input structure in checkmol-“tweaked” molfile format, it skips the entire ring search and aromaticity detection routines which saves considerable amounts of CPU time (see Results and Discussion). The matching process starts with the selection of a (preferentially) unique reference atom in the query structure by employing either a fast algorithm based on the degree of branching and the number of heteroatom substituents or using Morgan’s algorithm for canonicalization [28]. All possible counterparts of this reference atom in the candidate structure are enumerated and a recursive comparison of all substituents (match paths) in both structures is performed. By default, only local match matrices are used for each atom pair and all of their substituents, but also a global match matrix can be computed if required, e.g., if a complete atom/atom mapping of the query structure onto the candidate structure is desired (either for the first matching superposition or for all possible superpositions). In standard mode, atom pairs are compared for equivalence based on their chemical element, in “strict” mode also the subtypes (hybridization states) must match.

### 2.4. Structure Search Operation: Typical Procedure

A typical structure/substructure search operation in a structure data collection proceeds as follows: on the search page, the user draws the query structure in his/her web browser, using the JME applet. After selecting either the “exact” or “substructure” search option (additional options are “strict” and “check configuration”), the structure is submitted in molfile format to the web server. The receiving PHP script invokes checkmol via a shell call (using the PHP popen() function) or cmmmsrv via a TCP socket connection and passes the structure to the program. As the output of the latter, the molstat descriptors and the hash-based fingerprints are returned to the calling PHP script. In addition, the fragment dictionary is retrieved from the database and passed together with the query structure to matchmol (or cmmmsrv in matchmol mode). The resulting dictionary-based fingerprints of the query structure are returned to the PHP script. A special flag (*i.e.*, the least significant bit of the fingerprint bitstring, indicated by an odd number in its numeric representation) signals if the input structure happens to be identical with one of the dictionary fragments. If this is the case, the remaining search operation can be performed extremely fast by a single SQL statement without the need of candidate structure matching. In most cases, however, the full two-stage process comes into effect. This means, a first SQL query is issued with a list of molstat-derived conditions (e.g., number of C=O fragments equal or greater than 3, number of N-containing rings equal or greater than 1, *etc*.) in combination with fingerprint-derived conditions (e.g., bit number 5 in the dictionary-based fingerprint must be “1”, bits number 34, 57, 68, 99, *etc*. of the hash-based fingerprints must be “1”) as the search pattern. The latter type of search operation can be executed very efficiently by MySQL (and other relational database systems), using the “&” (“bitwise AND”) operator.

The resulting candidate structures are passed in appropriate portions together with the query structure (as the first element) to matchmol for full atom-by-atom comparison. The resulting stream of boolean information (“T” or “F”), together with a unique identifier, is then used by the PHP script for the assembly of the hit list. Hit structures are displayed in the browser either by using the JME applet in “depict” mode or – more efficiently – as bitmap images which have been rendered in advance (either during data import or later, by using the utility program mol2ps [29] in combination with the Ghostscript program [30]).

For similarity search operations, there is no need to perform atom-by-atom matching. Instead, the hash-based fingerprint of the query structure is used to compute the Tanimoto score [31] for each pairwise combination with a potential candidate’s hash-based fingerprint. For pre-selection, the molecular statistics descriptors (with adjustable margins) can be used as well as the number of “1” bits in the candidate fingerprints, as these numbers are already stored in the database table.

In the case of structure/substructure searches in a reaction data collection, cumulative dictionary-based and hash-based fingerprints are generated for all reactants and for all products of the query reaction, and these cumulative bitstrings (which are obtained by bitwise OR operations with individual fingerprints) are used to retrieve all possible candidate reactions by their cumulative fingerprints. Next, each reactant structure of the query is matched by matchmol against all reactants of a candidate reaction and each product structure of the query is analogously compared with all products of this candidate reaction. If all reactants and all products are found to be present in the candidate reaction, the latter is displayed as a hit or it is subjected to a final examination of atom/atom mappings, if they were specified in the query. This task is performed by the PHP script by making use of the maps which have been generated when the reaction was entered into the database.

## 3. Chemical Functionality as a Search Criterion

An option to retrieve chemical structures based on the presence of particular functional groups is not a common feature of structure databases, although it has been implemented in a few proprietary systems such as Chemical Abstracts Scifinder [32], the ChemDB portal of the Korea Institute of Science and Technology Information [33], or the NCI Screening Data 3D Miner of the University of Erlangen-Nürnberg [34]. To some extent, functional group searching can be substituted by substructure searching in conventional databases, but the result (apart from performance issues, see below) will not be exactly equivalent. For instance, an amine is characterized by at least one alkyl or aryl residue attached to a sp^3^-hybridized nitrogen and there must not be any heteroatom bonded to this nitrogen (such as in a hydrazine or a hydroxylamine) nor must there be an acyl residue on that nitrogen (such as in an amide or an imide). Thus, it is the combined presence *and* absence of certain groups of atoms which define a functional group. Such groups play an essential role in determining a molecule’s chemical reactivity/stability, its physicochemical properties (such as solubility), or its biological activity. They are clearly defined and therefore any information about chemical functionality of a compound can be un­ambigously communicated among chemists, in general. As shown previously, automatically assigned functional group classification can be extended into a chemical ontology [35].

With checkmol’s ability to recognize more than 200 functional groups in organic molecules, it is straightforward to add a functionality search option to structure and reaction databases. In the MolDB5R implementation, a special database table holds this information as a bitstring of 256 bits length, stored as 32-bit unsigned integer numbers. In a typical search operation, the user selects one or multiple functional groups from a clear-text list (implemented as a HTML form) and this search pattern is then translated into a query bitstring. By dividing the query bitstring into 32-bit segments in an analogous manner as it was done for the bitstrings in the database table, the SQL query string can be automatically composed, consisting of a series of conditions based on the “&” (“bitwise AND”) operator. In a simple example, a search for the following functionalities is executed:

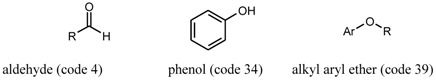


The resulting query bitstring would look like this (only the first 70 bits are shown here): “000**1**00000000000000000000000000000**1**0000**1**0000000000000000000000000000000…”. Then, the full 256-digit bitstring is split into eight 32-bit segments which correspond to the segments stored in columns *fg01, fg02, … fg08* in the database table. With bit positions numbered from 0 to 31 in each segment, the first two query segments and their decimal representation are now:

1) 000**1**0000000000000000000000000000 → bit number 3 is set, decimal value: **2^3^ = 8**
2) 0**1**0000**1**0000000000000000000000000 → bits 1 and 6 are set, decimal value: **2^1^ + 2^6^ = 66**

Based on the resulting two decimal integer numbers (up to 8 numbers are possible), the following example SQL query will retrieve all matching entries:

**SELECT ... WHERE (fg01 & 8 = 8) AND (fg02 & 66 = 66);**

Due to the highly efficient implementation of bitwise operators in modern relational database management systems, such queries are typically complete within fractions of a second even for larger data collections containing millions of entries.

In MolDB5R, also reaction databases offer the functional group search option. Here, cumulative bitstrings describe the functional groups detected in all reactants and in all products of a reaction, again they are stored as sets of 32-bit decimal numbers. Based on the separate treatment of reactants and products, the user can decide if the query selection of functional groups will be regarded as a) present in any reactant, b) present in any product, c) lost during the reaction, or d) created during the reaction. The latter two options require some additional SQL overhead, but again they are executed very fast.

## 4. Results and Discussion

In the following, some examples will be presented and discussed in order to demonstrate the scope and limitations of the developed software, with special attention to chemical functionality pattern matching as an additional search option in structure and reaction databases.

### 4.1. Data Import Performance: Speed versus Information Content

Whereas interactive entry of new structures or reactions into a data collection usually is uncritical with respect to processing speed, batch import of large numbers of entries can take a considerable amount of time, depending on the complexity of auxiliary information which is computed in order to facilitate efficient data retrieval. In order to estimate the computational “cost” of creating particular types of database information content, a series of batch import runs was performed on standard hardware (3.1 GHz Intel Core 2 Duo processor, 4GB of RAM), using 100,000 structures from the PubChem database as test sets.

**Figure 4:**
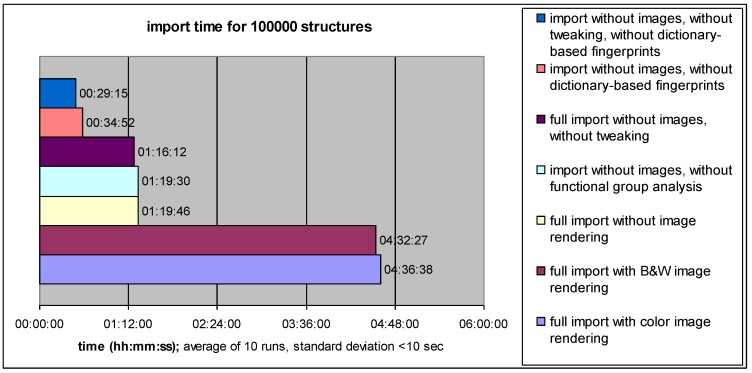
Time required for batch data import with different processing options.

As evident from Figure 4, generation of high-quality (anti-aliased) bitmap images of the 2D structure formulae by invocation of mol2ps and Ghostscript consumes considerable time resources, with a marginal difference between color and black-and-white images (using the png256 and pnggray Ghostscript drivers, respectively). Nevertheless, creation of bitmap images (either at import time or later on) offers the advantage of smooth and fast display of large hit lists without causing substantial CPU load on the client side (in contrast to embedding many instances of the Java applet on one page). If import time is critical, image rendering should be turned off and performed after completion of data import (bitmap graphics and JME-displayed vector images of structure formulae can co-exist within a hit list). 

Another major object of computational effort is the creation of dictionary-based fingerprints, which takes almost half of the remaining time (if image rendering is turned off). But also in this case, the investment appears justified in view of the significant performance gain which can be provided by these fingerprints, especially if a query structure happens to be identical with one of the dictionary structures (e.g., an unsubstituted ring system like benzene, pyridine, *etc.*). 

On the other hand, only subtle reductions in import time are gained when the “molfile tweaking” feature is omitted (1:16:12 *vs.* 1:19:46). As described in section 2.1., this type of aromaticity annotation can significantly save search time, as it prevents matchmol from repetitiously performing ring search and aromaticity detection routines for the structures already stored in the database.

As the most striking observation, an almost neglectable contribution of functional group analysis to the overall import time was found (1:19:30 *vs.* 1:19:46). This appears particularly remarkable in view of the powerful search operations which are made possible by this type of descriptors.

### 4.2. Search Performance

In order to evaluate the relationship between computational time investment during data import and search performance of the system, a series of test searches was carried out (Table 1 and Table 2) [36]. For this purpose, a structure database containing about one million entries which were imported from the PubChem database and a reaction database containing 500 entries which were excerpted from reactions published between 1999 and 2010 in the online journal *Molbank* (ISSN 1422-8599) [37] were used. 

The time required for completion of a query was measured at the server side by placement of the PHP function microtime() at the beginning and at the end of a code block performing the search operation. At the client side, additional delays (up to several seconds) may be observed which result from HTTP communication between the browser and the web server (especially if large numbers of structure formulae are to be displayed in a long hit list) as well as from cache-miss effects.

First, the effect of aromaticity annotation in stored structures (“tweaked” versus “untweaked” molfiles) was investigated. As can be seen from entries 1+2 and 3+4, substructure searches will benefit significantly from turning this option on during data import. For example, a substructure search for 5,11-dimethylpyrido[4,3-*b*]carbazole (the alkaloid *ellipticine*) gives 131 hits within 0.86 seconds with “tweaked” molfiles in the database, as compared to 2.30 seconds with unmodified molfiles (entries 3 and 4). Using caffeine as the query structure, 3,518 hits are regularly retrieved within 6.56 seconds, while the same query takes almost twice as long without this kind of acceleration. 

Although substructure searches and functionality searches cannot be directly compared, as they differ in generality/specificity, some examples are presented which give at least an impression of their performance in related tasks. For instance, sulfanilamide as the query structure in a substructure search (with explicit hydrogens for the arylamino group; entry 5) produces 905 hits within 13.46 seconds, in this case out of an untypically large number of 8,520 pre-selected candidate structures [38] 

**Table 1:** Comparison of performance between different search types and options in a database containing 1,043,008 structures.

Entry	Search type	Query	Remarks	Hits	Time (sec)
1	substructure	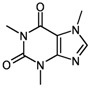 caffeine	with untweaked molfiles	3518 (of 3613 candidates)	11.20 (± 0.029)
2			–	3518 (of 3613 candidates)	6.56 (± 0.136)
3	substructure	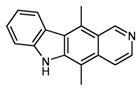 ellipticine	with untweaked molfiles	131 (of 216 candidates)	2.30 (± 0.007)
4			–	131 (of 216 candidates)	0.86 (± 0.010)
5	substructure	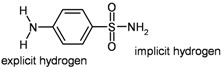	–	905 (of 8520 candidates)	13.46 (± 0.799)
6	functional groups	“primary aromatic amine” + “sulfonamide”	groups taken from entry 5	1912	0.16 (± 0.015)
7	substructure	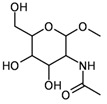	–	452 (of 591 candidates)	1.81 (± 0.029)
8	functional groups	“heterocycle” + “acetal” + “secondary carbox­amide" + “primary alcohol” + “secondary alcohol”	groups taken from entry 7	688	0.06 (± 0.001)
9	substructure	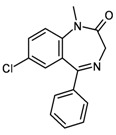 diazepam	–	127 (of 155 candidates)	0.48 (± 0.004)
10	functional groups	“aromatic” + “hetero­cycle" + “imine” + “tert. carboxamide” + “lactam” + “aryl chloride”	groups taken from entry 9	273	0.02 (± 0.000)

On the other hand, a functional group search for the pattern “primary aromatic amine” + “sulfonamide” (entry 6) gives 1,912 hits (which represent a superset of the hits from entry 5) in only 0.16 seconds. Entry 7 shows a substructure search for an *N*-acetyl-2-amino-2-deoxyaldohexose methyl glycoside, formulated in the pyranose form without any stereochemical definitions. The search yields 452 hits out of 591 candidate structures within 1.81 seconds, whereas the functional group search for the pattern defined by all of the functionalities present in this structure (“heterocyclic compound” + “acetal” + “secondary carbox­amide" + “primary alcohol” + “secondary alcohol”; entry 8) gives 688 hits in only 0.06 seconds. In the case of 7-chloro-1-methyl-5-phenyl-1,3-dihydro-1,4-benzodiazepin-2-one (diazepam; entry 9), the molecular graph is very well represented by the dictionary-based and hash-based fingerprints, a substructure search finds 127 hits (out of 155 candidate structures) within 0.48 seconds. Here, the functionality pattern (entry 10) is considerably less specific than the substructure query (273 hits), but again it is very fast, with a search time of only 0.02 seconds in a database of one million entries.

**Table 2:** Comparison of performance between different search types in a database containing 500 reactions.

Entry	Search type	Query	Remarks	Hits	Time (sec)
1	reaction substructure	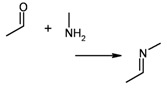	–	38 (of 56 candidates)	7.01 (± 0.004)
2	functional groups	“imine” created during reaction	group taken from entry 1	37	0.019 (± 0.000)
3	reaction substructure	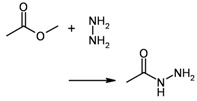	–	8 (of 20 candidates)	2.68 (± 0.002)
4	functional groups	“carboxylic acid hydrazide”created during reaction	group taken from entry 3	10	0.004 (± 0.000)
5	reaction substructure	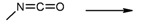 (half reaction)	–	10 (of 16 candidates)	0.73 (± 0.002)
6	functional groups	“isocyanate” lost during reaction	group taken from entry 5	10	0.005 (± 0.000)

In reaction databases, functional group patterns can also provide a very useful and efficient criterion for search operations, especially if a more general, method-oriented overview is desired. Table 2 lists three examples for queries (entries 1+2, 3+4, and 5+6), formulated both as reaction substructure and functional group searches. In these cases, the resulting hit sets are very similar, but again functionality matching is significantly faster. As a strength of reaction substructure searching, its higher degree of achievable specificity must be considered, particularly in combination with atom/atom maps.

## 5. Conclusions

With the MolDB5R package, including checkmol/matchmol as its cheminformatics engine, an easy-to-use and versatile open-source software for running a web-based structure/reaction database has been created and made freely available. Whereas structure/substructure search capabilities (also for reaction databases) cover the typical requirements of such systems, it could be shown that checkmol-assigned chemical functionality descriptors can add considerable value to the information content of both structure and reaction databases. Despite the very low computational “cost” for creating these fingerprints in addition to the other types of pre-selection descriptors, search operations based on functionality pattern matching are faster by at least one order of magnitude, they are easy to formulate (just by selecting well-defined terms from a list) and they can offer different, broader views at the contents of a chemical database.
